# Starvation vs. the “Ruling Passion”

**Published:** 1875-09

**Authors:** 


### STARVATION VS. THE "RULING PASSION."

An observer among the English factory girls describes their dinner-hour amusingly: The crowds had assembled outside of a certain cheap cook-shop in the half hour allowed for dinner. Among the girls was one ragged, scantily-clothed child of about fourteen. She stood for a long time wistfully before the cook-shop window. All the others had gone, and this forlorn object still stood there rattling a few half-pence in her hand. Finally, with a longing look at the precious display, she paused for a last sniff at the open door, and then dashed off down the street. The observer followed, thinking that she was seeking a cheaper cook-shop, and pitying her. But she stopped at a store where second-hand finery was for sale, entered, and in a few moments returned with a somewhat faded but still gorgeous bunch of artificial flowers, consisting of a rose full blown, a poppy or two, and a fair sprinkling of wheat. With a glow of triumph on her wizened face, she cast an eager glance to the right and left, and spying close at hand the secluded gateway of a timber yard, darted across the road, and crouching in a corner, was soon busy with her battered hat on her knees, trimming it.

---

Alum or vinegar is good to set colors—red, yellow or green.

---

A strong solution of carbolic acid and water poured into holes kills all the ants it touches, and the survivors immediately take themselves off.

---

If it be desired to cool water for drinking in warm weather, and ice can not be obtained, let it be kept in an unglazed earthenware vessel, wrapped around with two or three folds of coarse cotton cloth kept constantly wet.

